# Investigation of allosteric coupling in human β_2_-adrenergic receptor in the presence of intracellular loop 3

**DOI:** 10.1186/s12900-016-0061-9

**Published:** 2016-07-02

**Authors:** Canan Ozgur, Pemra Doruker, E. Demet Akten

**Affiliations:** Computational Science and Engineering Program and Polymer Research Center, Bogazici University, Istanbul, Turkey; Department of Chemical Engineering and Polymer Research Center, Bogazici University, Istanbul, Turkey; Department of Bioinformatics and Genetics, Faculty of Natural Sciences and Engineering, Kadir Has University, Cibali, 34083 Istanbul, Turkey

**Keywords:** β_2_-adrenergic receptor, Intracellular loop 3 (ICL3), G protein-coupled receptor, Allosteric coupling, Transmembrane helix

## Abstract

**Background:**

This study investigates the allosteric coupling that exists between the intra- and extracellular parts of human β_2_-adrenergic receptor (β_2_-AR), in the presence of the intracellular loop 3 (ICL3), which is missing in all crystallographic experiments and most of the simulation studies reported so far. Our recent 1 μs long MD run has revealed a transition to the so-called *very inactive* state of the receptor, in which ICL3 packed under the G protein’s binding cavity and completely blocked its accessibility to G protein. Simultaneously, an outward tilt of transmembrane helix 5 (TM5) caused an expansion of the extracellular ligand-binding site. In the current study, we performed independent runs with a total duration of 4 μs to further investigate the *very inactive* state with packed ICL3 and the allosteric coupling event (three unrestrained runs and five runs with bond restraints at the ligand-binding site).

**Results:**

In all three independent unrestrained runs (each 500 ns long), ICL3 preserved its initially packed/closed conformation within the studied time frame, suggesting an inhibition of the receptor’s activity. Specific bond restraints were later imposed between some key residues at the ligand-binding site, which have been experimentally determined to interact with the ligand. Restraining the binding site region to an open state facilitated ICL3 closure, whereas a relatively constrained/closed binding site hindered ICL3 packing. However, the reverse operation, i.e. opening of the packed ICL3, could not be realized by restraining the binding site region to a closed state. Thus, any attempt failed to free the ICL3 from its locked state due to the presence of persistent hydrogen bonds.

**Conclusions:**

Overall, our simulations indicated that starting with *very inactive* states, the receptor stayed almost irreversibly inhibited, which in turn decreased the overall mobility of the receptor. Bond restraints which represented the geometric restrictions caused by ligands of various sizes when bound at the ligand-binding site, induced the expected conformational changes in TM5, TM6 and consequently, ICL3. Still, once ICL3 was packed, the allosteric coupling became ineffective due to strong hydrogen bonds connecting ICL3 to the core of the receptor.

**Electronic supplementary material:**

The online version of this article (doi:10.1186/s12900-016-0061-9) contains supplementary material, which is available to authorized users.

## Background

Human β_2_-adrenergic receptor (β_2_-AR) is a member of the G-protein coupled receptor (GPCR) superfamily that is responsible in the eukaryotic signal transduction, responding to hormones adrenaline and noradrenaline to mainly induce the smooth muscle relaxation in the lung tissue. As all members of GPCRs, β_2_-AR shares the 7TM structural motif, which consists of seven transmembrane-spanning alpha helices connected by loop regions at the intra- and extracellular sides of the membrane.

As the first hormone-activated GPCR structure to be reported by X-ray crystallography [1, 2], the high resolution structural information was obtained through the elimination of the third intracellular loop (ICL3) replaced with the protein T4 lysozyme (T4L) and also the C-terminal tail in order to increase both the proteolytic stability and crystallizability. ICL3 links the cytoplasmic ends of transmembrane helices V and VI (TM5 and TM6) and has a functional role for both the recognition and the activation of G proteins [3, 4]. Unlike other intracellular loop regions, ICL3 has a highly variable length and sequence among the members of the GPCR superfamily, even the closely related subtypes. It is believed that a GPCR’s selectivity to different G proteins originated in the structural uniqueness of ICL3.

One experimental study conducted by West et al. [5] investigated the ligand-dependent perturbation of the conformational ensemble for β_2_-AR, which incorporated the ICL3 region through hydrogen/deuterium exchange (HDX) coupled with mass spectroscopy. HDX data suggested ICL3’s role as a molecular switch, where antagonist and inverse agonist binding shifted the equilibrium toward inactive states, which is characterized by protection to exchange (i.e., stabilization) in the ICL3 loop and flanking regions of TM helices V and VI. In contrast, binding of a full agonist shifted equilibrium toward higher accessibility and/or destabilization of ICL3 region. The lack of mobility or the stabilization of ICL3 was also observed in our previous work by Ozcan et al.[6], where the presence of ICL3 had a significant impact on the overall dynamics of the receptor, especially the arrangements of some key residues at both the ligand-binding site and the G-protein binding site. In that study, in addition to the *loop* model where the missing ICL3 region was generated in fully atomistic detail, a second model called *clipped* model was created with the two open ends of TM5 and TM6 covalently bonded to each other. During the 1 μs long MD run, the *loop* model found a very inactive conformation towards 600 ns, when TM6 moved towards the receptor’s core region with ICL3 packing underneath the membrane and blocking the G-protein’s binding site. ICL3 preserved its closed conformation and consequently, the receptor’s overall dynamics has decreased significantly with only minor fluctuations for the remaining 400 ns, and became similar to the *clipped* model’s dynamics, which showed no major variations.

Early experimental peptide studies showed that the peptides synthesized with the primary sequence 259–273 corresponding to ICL3 region of G_s_-coupled β_2_-AR, selectively bind with G proteins, stimulate their functional activity, trigger signaling cascade in the absence of hormonal stimulus, and decrease the regulatory effects of β_2_-AR agonists [7–9]. Another peptide study revealed that the ICL3-derived peptides can form helical structures and contain clusters of positively charged residues exposed to one helix side which is crucial for interacting with the negatively charged receptor binding site of the Gα_s_ subunit (Shinagawa et al. [10], Okuda et al, [11]). The last result is in good agreement with the simulation study by Ozcan et al. [6] during which the unstructured loop region generated through modeling techniques adopted a few turns of helices before blocking the G-protein binding cavity.

Yet, despite its functional significance, many experimental and simulation studies conducted so far have neglected its presence [12–15]. The present work focuses on the effect of the intracellular loop region ICL3 on the intrinsic dynamics of the receptor. The intrinsic dynamics is the key determinant of the receptor’s function, whereas the tertiary structure encodes the dynamic behavior. Thus, it is important to have a complete 3D structure of the receptor in order to understand the system’s function to a greater degree.

The study presented here adopted the same atomistic model of the receptor that incorporates the ICL3 region generated in our previous study [6]. First, we start by investigating the stability of the alternative inactive state of the receptor, which was observed during the last half of the 1 μs long MD simulation. The most distinguishing features of the alternative state was the closure of ICL3 that completely blocked the G protein binding site at the intracellular region, and the simultaneous enlargement of the ligand-binding site at the extracellular part. Both regions of the receptor changed their conformation almost simultaneously which suggested a strong allosteric coupling. In two independent continuation MD runs (500 ns long each), the receptor preserved its stable ICL3 conformation as well as the extracellular part of the receptor.

In addition to the presence of ICL3, the allosteric effect was further investigated through specific bond restraints between key residues at the ligand-binding site, which led to an alternative closed state. Also, the probability of open-to-closed or closed-to-open transition in ICL3 conformation was revealed for the first time. Addition of restraints mimics the presence/interaction of the ligand and possibly accelarates the closure event. So, the conformational shift that would take place in the presence of the ligand would require much longer simulations, whereas the restraints have provided an enhanced event sampling by providing an exaggerated driving force or perturbation at the binding site.

Even though the allosteric coupling that exists between the intracellular and the extracellular regions of the receptor is a well-known feature of β_2_-AR and many other GPCRs, the presence of ICL3, which directly influences the overall dynamics of the receptor, was not taken into consideration when the coupling behavior was investigated. Therefore, the present study will be the first in providing this correlated motion that exists between the extracellular ligand-binding site and the intracellular G-protein binding site that incorporates the ICL3 region. Through imposing distance restraints between some key residues at the ligand-binding site, we were able to trigger a series of conformational changes in the transmembrane helices that led to the close packing of ICL3.

## Methods

### Protein-membrane system preparation

The system under study was adopted from Ozcan’s work [6] where the initial conformation was the x-ray crystallographic structure of human β_2_AR in complex with T4 lysozyme and the partial inverse agonist carazolol (PDB id: 2RH1) at 2.4 Å resolution [2]. The anchor protein T4L was covalently attached to two ends of helices 5 and 6 in order to facilitate crystallization. After removal of T4L, the missing intracellular loop region ICL3 was added between residues 230 and 262 after being modeled as an unstructured loop of 32 residues long via homology modeling tool, MODWEB [16]. Also, the bound ligand at the ligand-binding site was removed and the apo form of the receptor was used as an initial conformation.

The receptor was then embedded in the double-layered 1-palmitoyl-2-oleoyl-phosphatidylcholine (POPC) phospholipid cell membrane generated with VMD’s Plug-in tool [17]. The receptor was positioned with an oblique angle of 8° between its main principal component along the membrane and the z-axis [18]. After solvating the protein-lipid system with VMD’s solvate module, Na^+^ and Cl^-^ ions were added to neutralize the total charge of the system, which is necessary for Particle-Mesh Ewald summation method used in electrostatic energy calculations [19]. The resulting periodic box dimensions were (86x86x100) in Angstrom. The total number of atoms in the system was 68.001, of which 5.055 belong to protein, 20.770 to lipids, 42.135 to water molecules, and 41 to ions.

The system’s equilibration consisted of several stages such as melting of lipid tails, minimization and equilibration with protein restrained, equilibration with protein released and lastly the production runs [6]. Nanoscale Molecular Dynamics (NAMD) v2.8 software tool was used for all our MD runs [20]. The force fields used were CHARMM27 [21, 22] for lipids, CHARMM22 [23, 24] for proteins and TIP3P model for water in the system [25]. In this work, the isothermal-isobaric (NPT) ensemble was employed using Langevin dynamics in order to keep the temperature constant with a Langevin damping coefficient (gamma) of 5/ps for all non-hydrogen atoms. The pressure was kept constant at 1 atm using a Nose-Hoover Langevin piston with 100 fs period and 50 ps damping timescale [26]. Long-range electrostatic interactions were treated by particle mesh Ewald (PME) method, with a grid point density of over 1 Å. A cutoff of 12 Å was used for van der Waals and short-range electrostatics interactions with a switching function. Time step was set to 2 fs by using SHAKE algorithm for bonds involving hydrogens [27] and the data was recorded at every 200 ps. The number of time steps between each full electrostatics evaluation was set to 2. Short-range non-bonded interactions were calculated at every time step.

### Independent MD runs with and without restraints

All MD runs are listed in Table 1. The first one is actually from our previous study and will be used as reference here. Two independent 500 ns runs named as “*MD1μs_cont1”* and “*MD1μs_cont2”* were continuations of the first run. They were based on the final snapshot of the original run “*MD1μs”*, which corresponds to a *very inactive* state of the receptor with a closed ICL3 that packed underneath the receptor [6]. No restraints were applied to the binding site in these continuation runs. The goal here was to observe how long ICL3 would preserve its closed state, which also indicate its conformational stability.

**Table 1:** Details of several MD runs with and without restraints

Run#	Run Name	Total time (ns)	Restrained (Yes/No)	Initial structure
1	*MD1μs* ^a^	1000	No	inactive crystal structure, 2RH1
2	*MD1μs_cont1*	500	No	last frame of *Run #1*
3	*MD1μs_cont2*	500	No	last frame of *Run #1*
4	*rstr1*	500	Yes	initial frame of *Run #1*
5	*rstr2*	500	Yes	initial frame of *Run #1*
6	*rstr3*	500	Yes	frame @ 470^th^ ns of *Run #1*
7	*rstr4*	500	Yes	frame @ 470^th^ ns of *Run #1*
8	*MD500ns*	500	No	last frame of *Run #7* (*rstr4*)
9	*rstr5*	500	Yes	last frame of *Run #1*

^a^This run was performed prior to this work in Ozcan’s study [6]. The remaining runs #2 through #10 were based on the same system created for run #1

In order to study the effect of restraints on the overall dynamics of β_2_AR, especially of ICL3, additional bond energy terms were applied to seven pairs of key residues located at the ligand-binding cavity that are known to be critical in binding signaling molecules (See Table 2). All extra bonded terms were harmonic potentials of the form *U*(*x*) = *k*(*x* − *x*_*ref*_)^2^ where *k* is the spring constant and *x*_*ref*_ is the restraint distance value. A total of five independent MD runs named as *rstr1 to rstr5,* under different restraints and with different starting conformations were performed as listed in Table 1*.* One additional 500 ns long MD run was performed on the last snapshot of *rsrt4* (Run #8) with all distance restraints removed in order to investigate the stability of the packed conformation of ICL3 in a restraint-free environment.

**Table 2:** Restraint distances in all seven MD runs and their corresponding values in experimentally reported active and inactive states

		Distances in crystallographic structures (Å)		Bond Restraints (Å)				
Residue pair	Exper.^b^ (Å)	Inactive (PDB id: 2RH1)	Active (PDB id: 3SN6)	*rstr1*	*rstr2*	*rstr3*	*rstr4*	*rstr5*
Ser203O^γa^-Asp113C^γa^	8.0–10.0	11.2	10.3	17	8	17	17	8
Ser204O^γ^-Asp113C^γ^	8.0–10.0	14.2	12.4	14	10	14	14	10
Ser207O^γ^-Asp113C^γ^	8.0–10.0	11.5	10.4	11.7	8	11.7	11.7	8
Ser207C^α^-Asp113C^α^	N/A	12.2	12.0	-	-	-	17	-
Asn293C^β1^-Asp113C^β^	8.0–10.0	13.6	14.0	14	15	14	14	8
Phe289C^β^-Asp113C^β^	8.0–8.4	11.7	12.3	13	12	13	13	8
Asn312 C^β^-Asp113C^β^	8.0–8.4	9.1	8.6	10	9	10	10	8
Phe289C^β^-Asn312C^β^	8.0–8.4	5.5	5.5	5.5	5.5	5.5	5.5	8

^a^γ Oxygen, and β and γ Carbon atoms of the side chains were taken into consideration

^b^These are the distance ranges observed previously in various experimental studies [28–33]

**Fig. 1:**
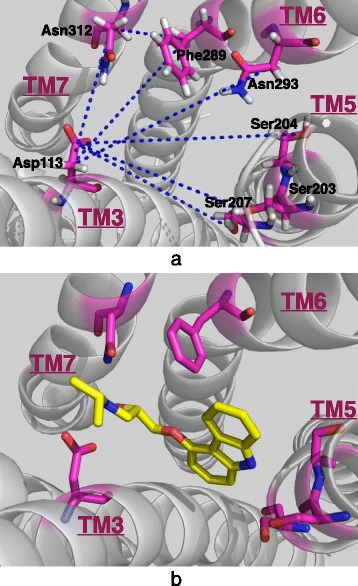
Extracellular view of the ligand-binding site. **a** Only key residues and the seven restrained distances are highlighted (**b**) From the same angle as in (**a**), the ligand carazolol as bound in the crystal structure of the inactive state (PDB: 2RH1)

The restraint distances that we selected for restraint MD runs are provided in Table 2. Seven of these distances are between Asp113 on TM3 and residues on TM5 (Ser203, Ser204, Ser207), TM6 (Phe289, Asn293) and TM7 (Asn312). One other is between residues on TM6 and TM7. Mainly, the size of the ligand-binding site is determined by the position of TM5, TM6 and TM7 with respect to the more stationary TM3 as depicted in Fig. 1. The distance ranges previously observed for the active state of the receptor in several different experimental studies [28–33] are also listed in the second column.

Table 2 also lists the same distances in both active and inactive x-ray structures (PDB ids: 3SN6 and 2RH1). For the inactive state, all distances are higher than the experimentally observed ranges, as expected. Surprisingly, for the active state, almost all distance values are slightly out of range. Yet, most of them are smaller than those in the inactive state, especially those between three serine residues on TM5 and Asp113 on TM3. Therefore, we will focus on the first three distances (rows in Table 2), which clearly distinguish the active states from the inactive ones.

In the first restrained MD run (*rstr1*), Ser203-Asp113 distance was set to 16 Å for the first 300 ns and then increased to 17 Å for the remaining 200 ns. Other distances were set to values closer to those observed in the inactive crystal structure. Here, the value of 17 Å was explicitly selected as it was recorded in the last frame of the original MD run (*MD1μs*), when ICL3 closed upon G-protein binding site.

In the second restrained MD run (*rstr2*), the same three critical distances between serine residues and Asp113 were restrained to 8 Å, 10 Å and 8 Å, which all fall within the experimental range of the active state. Similarly, the other distances were set closer to those in the crystal structures. The third run (*rstr3*) almost share the same set of restraints as in *rstr1* and *rstr2*, except that it starts with a different initial conformation of the receptor (See Table 1). In the fourth run (*rstr4)*, the same initial conformation was used as in *rstr3,* with an additional restraint distance set between two backbone alpha-carbon atoms in Ser207 and Asp113. Finally, in the last run (*rstr5*), the final frame of the original MD run (*MD1μs*) was used as a starting conformation and the ligand-binding site was severely constricted.

## Results and discussion

### Analysis of two continuation runs indicates stability of packed ICL3

**Fig. 2:**
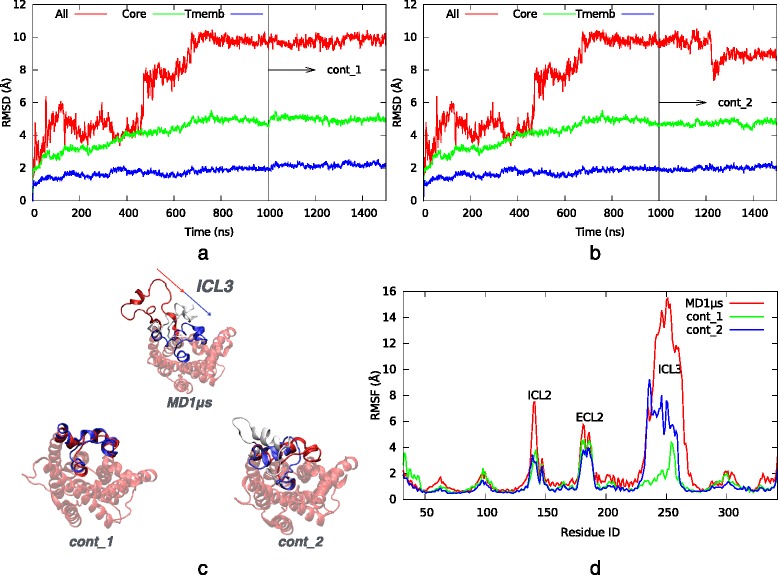
Results of original and continuation runs without restraints. RMSD profiles of (**a**) the first *MD1μs_cont1* and (**b**) the second *MD1μs_cont2* continuation runs (500 ns each) shown together with the *original* 1 *μs* MD run. **c** Intracellular view of the initial (red), intermediate (white) and final (blue) snapshots superimposed for each run. **d** RMSF profiles for the *original, MD1μs_cont1* and *MD1μs_ctd2* runs

Two 500 ns long MD runs were performed as an extension of the original *MD1μs* simulation (Ozcan et al.*,* [6])*,* as previously described in Methods section. Initially in *MD1μs* run, ICL3 was in an extended conformation, and highly mobile as illustrated in the upper portion of Fig. 2c (red ribbon). At around 600 ns of *MD1μs*, it started to pack under β_2_AR and kept its stationary state until the end of the simulation. The aim here was to investigate how long this stationary, and relatively restricted state would carry on. Both extended simulations, the so-called *MD1μs_ctd1* and *MD1μs_ctd2,* selected the final snapshot of the original run as their initial conformation. *MD1μs_ctd1* started with the same velocities as in *MD1μs’*s final state, whereas *MD1μs_ctd2* was carried out with a different velocity distribution, in order to enhance the sampling.

During the first continuation run, ICL3 stayed in its close form with only minor fluctuations (~2 Å) as shown in the RMSD profiles illustrated in Fig. 2a. All the RMSD calculations were carried out after the alignment of each MD snapshot in the trajectory to the initial snapshot based on the transmembrane region, as it is the least mobile part of the receptor. The RMSD profiles labeled as *All, Core,* and *Tmemb* represent the RMSD of the whole receptor, the core which is the receptor without ICL3 and the transmembrane region which consists of seven alpha helices located inside the membrane, respectively (See Additional file 1: Figure S1). This complete blockage of the G protein’s binding site suggests an inhibition of the receptor’s activity.

In the second continuation run using a different velocity distribution, a temporary increase up to 5 Å in the RMSD value was observed halfway through the trajectory, which led to a slight opening of ICL3 as reflected by the white colored snapshot in Fig. 2b. However, this opening was only temporary and lasted for about 20 ns. This was followed by a sharp decrease in RMSD to 4 Å caused by the closure of ICL3 to a position slightly different than the initial state. ICL3 stayed there for the rest of the simulation.

In both continuation runs, the closed state of ICL3 was preserved, representing an extreme inactive state, where the G-protein binding site was completely blocked. Experimental studies revealed both active and inactive states of the receptor, but none of these structures incorporated the ICL3 region. Here, it is the presence of ICL3 that caused the receptor to adopt such a novel inactive state, which was found to be noticeably stable.

The fluctuation of each residue averaged over the whole trajectory was determined for each simulation and illustrated in Fig. 2d. As expected, in both continuation runs, the mobility of ICL3 stayed at a much lower level than in the original MD simulation. Moreover, a slight decrease of mobility was observed in every part of the receptor, especially on two important loop regions, ICL2 (intracellular) and ECL2 (extracellular), in conjunction with the decrease in ICL3’s mobility.

**Fig. 3:**
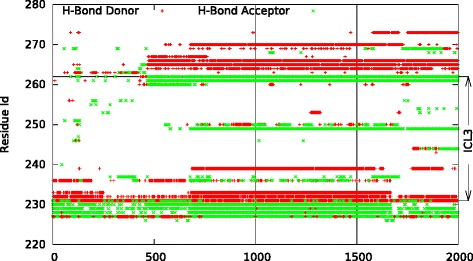
Hydrogen bond profiles. The first and the second continuation runs (500 ns each), MD1ms_cont1 and MD1ms_cont2, covering a time range of [1000:1500] and [1500-2000] ns, respectively. The first 1000 ns corresponds to the original run. Donor and acceptor groups are illustrated by red and green dots, respectively

The stability of ICL3 was further investigated by a detailed analysis of the hydrogen bond network in the loop conformation. Figure 3 illustrates the profile of the residues involved in hydrogen bonding along the trajectory, which was focused on ICL3 and its neighborhood region. By the time ICL3 closure is completed at around 600–700 ns, a total of 8 stable hydrogen bonds has been observed between ICL3 and the rest of the receptor (core region), which was maintained throughout the simulation. It is noteworthy that this stable network of hydrogen bonds was located mostly at the two junctions of ICL3-TM5 and ICL3-TM6. In the first continuation run, nearly all 8 hydrogen bonds were preserved, whereas in the second continuation run, half of them was lost when a slight opening was observed, but still, an alternative close state of ICL3 was adopted later towards the end of the simulation with most of the hydrogen bonds recovered.

In our simulation studies, the closure of ICL3 was strongly coupled with the lower part of TM6, which exhibited an inward movement of 7.5 Å, in the opposite direction of the outward movement of 14 Å observed during activation (experimentally measured at the Cα carbon of Glu 268 [1]). The RMSD profiles of the intracellular part of TM6 with respect to the active state (PDB id:3SN6) illustrated at the top section of Additional file 2: Figure S2 show that as ICL3 started to change its conformation to a closed state, TM6’s intracellular part shifted to the opposite direction of activation and stayed there for both continuation runs. On the other hand, the intracellular part of TM5 attached to ICL3 at the other end, seemed unaffected by these conformational variations. As illustrated at the lower section of Additional file 1: Figure S1, TM5 stabilized at around 2 Å during the original MD as well as both continuation runs.

Two of the key residues at the binding site are Asp113 on TM3 and Ser207 on TM5, which are known to interact both with agonists and antagonists, via hydrogen bonds or close contacts. They are situated at the two distant corners of the binding site and when the ligand is favorably bound, Ser207 is near the ligand’s aromatic moiety, while Asp113 usually makes multiple hydrogen bonds with the ligand’s polar end group (See Fig. 1b). Therefore, the distance between these two residues directly controls the binding capability of the ligand. Experimental measurements already determined an approximate distance range of [8 Å -10 Å] between the two side chain atoms, O^γ^ of Ser207 and C^γ^ of Asp113, when the receptor was found in its active state [31, 32]. As the receptor passes from an active state to an inactive one, the same distance also increases and stabilizes roughly at around 11 Å -12 Å. Thus, as an indicator of activation/inactivation, the same distance was monitored for both continuation runs.

**Fig. 4:**
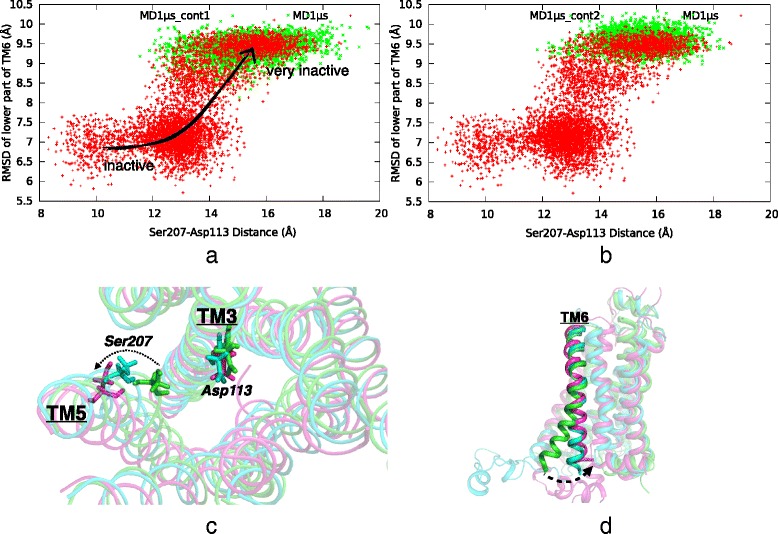
Simultaneous conformational changes in extracellular and intracellular parts of the receptor. **a**, **b** RMSD of intracellular part of TM6 with respect to active state (PDB id: 3P0G) vs. distance between Ser207-O^γ^ and Asp113-C^γ^ in two continuation runs (green dots) together with the original *MD1μs* run (red dots). **c** Extracellular view of the binding site and **d** side view of TM6 for which the active state, initial and final snapshots of *MD1μs* run were colored in green, blue and magenta, respectively

In our *original* MD run, a close correspondence between this value and the conformational state of the lower part of TM6 was established as shown in Fig. 4; as ICL3 exhibited its major conformational shift from an open to a closely packed state, the lower part of TM6 shifted towards the core of the receptor (See Fig. 4d) and at the same time, the Ser207-O^γ^ and Asp113-C^γ^ distance started to increase up to 17 Å - 18 Å, which is majorly caused by the outward shift of TM5 (See Fig. 4c). In both continuation runs, the same distance fluctuated within a range of 13 Å - 18 Å, which is still above 11 Å -12 Å of the crystal structure of the inactive state [1, 2] (See Fig. 4a and b). Moreover, no significant conformational change in the lower part of TM6 was observed, which is mainly caused by the stationary ICL3.

### Rapid closure of ICL3 is observed as restraints expand the ligand-binding site

The goal here was to investigate the allosteric coupling between the intra- and extracellular parts of the receptor, by applying some distance restraints to several key residues at the extracellular ligand-binding site region (See Fig. 1). As listed in Table 1 (See Methods section), there exist seven distances between side chain atoms that were experimentally observed within a certain range when the receptor adopted an active state [28–33]. In our first constrained simulation (*rstr1*), one of those distances which exists between S203O^γ^ and D113C^γ^, was restrained to 16 Å for 300 ns and later increased to 17 Å for another 200 ns, while the remaining six were restrained to those observed in the inactive crystal structure (PDB id: 2RH1) for the whole 500 ns long simulation (See Table 2).

The high value of 17 Å was especially selected for S203O^γ^-D113C^γ^ distance in order to reveal the same allosteric response of the intracellular part of TM6 and ICL3 observed previously in the original MD run. As expected, a close correspondence was observed between the extracellular and intracellular parts of the receptor, as ICL3 packed towards the core of the receptor by the end of 200 ns, which is about 400 ns earlier than in the original *MD1μs*.

**Fig. 5:**
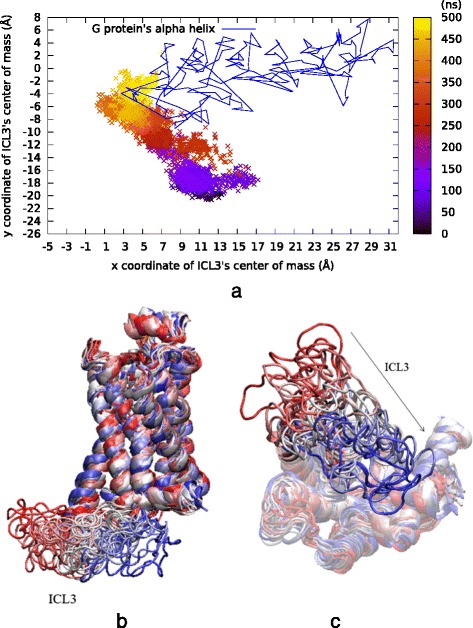
Results of 500 ns long *rstr1* run. **a** ICL3’s center of mass (*x* and *y* only) color-coded by time step. Lines represent the G protein’s α helix *x* and *y* coordinates extracted from the active state’s crystal structure (PDB id: 3SN6). **b** Side and **c** intracellular views of 20 snapshots colored from red (initial), to white (intermediate), to blue (final) during simulation

The closure of ICL3 was monitored through the *x* and *y* coordinates of its center of mass, as illustrated with colored points corresponding to different time regimes in Fig. 5a. The interacting alpha helical part of G protein was shown as a straight line connecting all its *x-y* coordinates, simply to give an idea about its position with respect to ICL3. Also, in Fig. 5b and c, a total of 20 snapshots gradually changing color from red to white and finally to blue well demonstrate the closure of ICL3 towards the core of the receptor during simulation in different angles.

### ICL3 preserves its open conformation as restraints narrow the ligand-binding site

**Fig. 6:**
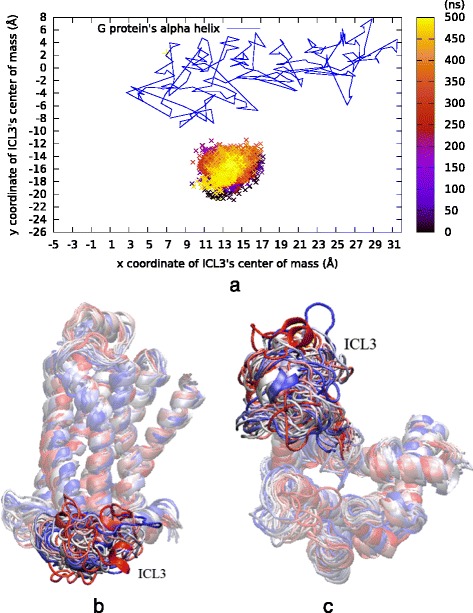
Results of 500 ns long *rstr2* run. ICL3 preserves its initial open state as bond restraints narrows the ligand-binding site, in agreement with the allosteric coupling behavior between the intra- and extracellular parts of the receptor. **a** ICL3’s center of mass (x and y only) color-coded by time step. Lines represent the G protein’s a helix x and y coordinates extracted from the active state’s crystal structure (PDB id: 3SN6). **b** Side and (**c**) intracellular views of 20 snapshots colored from red (initial), to white (intermediate), to blue (final) during simulation

A second restrained MD run (*rstr2*) was performed with the same initial frame as used in the first run. This time, the ligand-binding site region was narrowed down via bond restraints between three serines (Ser203-O^γ^, Ser204-O^γ^, Ser207-O^γ^) and Asp113-C^γ^ to 8 Å, 10 Å and 8 Å, respectively. The simulation was performed for a total of 500 ns. ICL3 preserved its initially open conformation throughout the simulation, in agreement with the allosteric coupling behavior between the intra- and extracellular parts. Similar to the first restrained run, the position of ICL3’s center of mass was monitored and all 20 snapshots were illustrated from the side and the intracellular views as in Fig. 6.

### ICL3 closure necessitates the outward tilt of TM5

One important finding about the closure of ICL3 in the first restrained run and also the original run was the simultaneous outward tilt of TM5 towards the lipid bilayer, which is crucial in initiating the conformational changes along TM5 and TM6 and consequently on ICL3 (See Fig. 4c). In both runs, the distance restraints applied to residues on TM3 and TM5 shifted TM5 but not the more stationary TM3. Consequently, this desired outward tilt in the extracellular part of TM5 was followed by the inward tilt of the intracellular part of TM5 and also of TM6, which induced the expected ICL3 closure (See Fig. 4d).

The necessity of TM5’s outward tilt was demonstrated in another 500 ns long restrained run (*rstr3* in Table 2) that used similar restraints as in the first restrained run, but an alternative initial conformation, in which ICL3 was in an extended form but slightly packed and oriented towards the core of the receptor (See Additional file 3: Figure S3). The applied restraints simply necessitated an *expanded* ligand-binding site, which was expected to induce the closure of ICL3. However, no closure was observed in ICL3, which covered a wide range of alternative states nearby G-protein binding site and towards the end of 500 ns, ended up close to its initial position (See Additional file 4: Figure S4). When the conformational change in TM5 was observed, it was clear that as a result of the distance restraint, the outward tilt in TM5 was not notable since both TM3 and TM5 moved apart at the extracellular side (See Additional file 5: Figure S5). Furthermore, no major conformational change in the intracellular part of TM5 was observed. Consequently, ICL3’s motion stayed random between open and close states, and no closure was observed. This result shows that the inward tilt of TM6 at the intracellular side was not enough to induce the closure of ICL3, which necessitates the inward tilt in both TM5 and TM6.

**Fig. 7:**
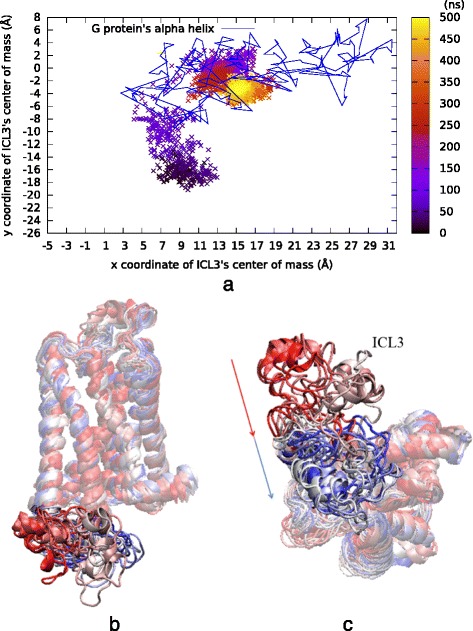
Results of 500 ns long *rstr4* run. The expected ICL3 closure was observed under 100 ns, when an additional bond restraint was imposed between the backbone atoms in the ligand-binding site. **a** ICL3’s center of mass (x and y only) color-coded by time step. Lines represent the G protein’s a helix x and y coordinates extracted from the active state’s crystal structure (PDB id: 3SN6). **b** Side and (**c**) intracellular views of 20 snapshots colored from red (initial), to white (intermediate), to blue (final) during simulation

Our next attempt in *rstr4* was to impose an additional bond restraint that will bring out the desired outward tilt in TM5, which was not obvious in our previous run (*See* Table 2). Since the backbone atoms’ fluctuations are usually minor compared to those of side chain atoms, the bond restraint of 17 Å was imposed between two backbone atoms, C^α^ atom of Ser207 and C^α^ atom Asp113. This time, the new additional restraint was expected to cause the important outward tilt in the extracellular part of TM5. Indeed, both the expected ICL3 closure and the desired outward tilt in TM5 were observed. In addition, ICL3 closure was accomplished under 100 ns, which was two times faster than the first restrained run (See Fig. 7). Another difference was the final position of ICL3, which was shifted about 5 Å in the *x*-axis with respect to the previously observed positions and located towards the middle of the G-protein binding cavity. In order to further investigate the stability of ICL3 in this alternative closed state, another 500 ns long MD run (*MD500ns)* was performed with all restraints removed (*run #8* in Table). ICL3 preserved its closed state as illustrated with the center of mass profile in Additional file 6: Figure S6.

### Packed ICL3 could not be opened by constricting the ligand-binding site

**Fig. 8:**
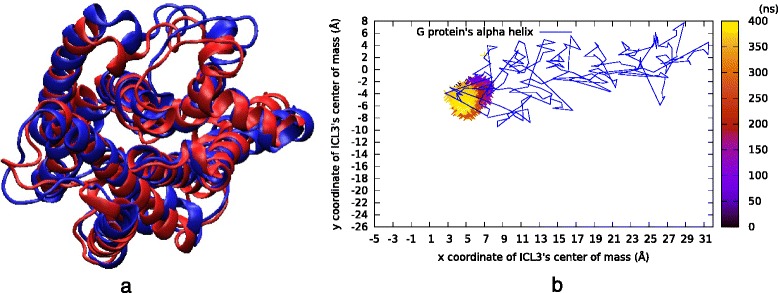
Results of the fifth restrained *rstr5* run. Here, the bond restraints narrowed the ligand-binding site region while ICL3 was completely packed. The initially packed/closed state of ICL3 was preserved throughout 500 ns long simulation. **a** The initial (red) and final (blue) snapshots of MD run, (**b**) ICL3’s center of mass (x and y only) color-coded by time step, where lines represent the G protein’s a helix x and y coordinates extracted from the active state’s crystal structure (PDB id: 3SN6)

The final restrained run (*rstr5*) was set up to observe the allosteric effect caused by narrowing the ligand-binding site region. The final snapshot of the original MD run (*MD1μs*) was taken as the initial conformation. Here, the ICL3 was fully packed, blocking the G-protein binding site. The ligand-binding site was severely constricted with distance values of 8 Å between almost all pairs of atoms (See Table 2), yet any attempt failed to free the ICL3, which only covered a very confined space during 500 ns long MD run (See Fig. 8). This last result simply point to an important aspect of the receptor’s dynamics. It is rather easy to induce the packing of a loose ICL3 by expanding the extracellular binding site region. Yet, it is almost impossible to unpack an already packed or a half packed ICL3 by simply narrowing the extracellular binding site region. Clearly, the energetic barrier to unpack the ICL3 and consequently open the G-protein binding site is too high to be overcome by a few restraints applied at a far region of the receptor. This energetic barrier is most likely due the existence of several hydrogen bonds that exist between ICL3 and the adjacent ends of TM5 and TM6. Thus, the outward tilt of TM6 including the ICL3, which exposes the G-protein binding site, needs to be induced by some exterior forces acting directly on that specific region only.

One activation mechanism proposed by Dror et al. [34] also supports this finding. They have shown that in its basal form, the receptor’s intracellular part of TM6 fluctuated between open or half open (intermediate) states, and adopted a fully open active state only when a G protein was bound from the intracellular region and pushed the binding site to an open form. If an agonist was bound at the extracellular binding site, then this active state was stabilized. On the other hand, when G protein was released from this agonist-bound state, it was observed that the receptor’s intracellular part of TM6 quickly returned to its inactive state obstructing the G-protein binding site. This finding indicated that the active state cannot be induced by some agonists alone and can only be reached by some exterior forces.

## Conclusions

The very inactive state of the receptor [6], was further investigated by two 500 ns long MD runs, which presented a highly stable packed state of ICL3. Although a slight tendency for its opening was observed in one of these simulations (Fig. 2), the closed state was adopted shortly afterwards. The hydrogen bond network analysis revealed several hydrogen bonds connecting ICL3 with adjacent TM5 and TM6 regions, thus stabilizing this novel packed state.

Several distance restraints were applied to key residues at the extracellular ligand-binding site in order to investigate their effect on the intracellular G-protein binding site including ICL3. Bond restraints caused either an expansion or constriction of the ligand-binding site. Key distances that majorly control the size of the cavity were between Asp113 on TM3 and three serine residues (S203, S204 and S207) on TM5. When the ligand-binding site was forced to an expanded/open state via these restraints (*rstr1*), ICL3 closure took place following a straight pathway towards the G-protein binding site, as in the original *MD1μs* run. On the other hand, when the same ligand-binding site was forced to a constricted state (*rstr2*), no change at the intracellular part was observed as ICL3 preserved its initial open conformation. These two observations were both in agreement with the ‘pincer-like’ behavior of the receptor, where the intracellular part becomes wider as the extracellular part becomes narrower, and vice versa [35].

In both runs *MD1μs* and *rstr1*, closure of ICL3 was observed to closely couple with the inward tilt of both TM5’s and TM6’s intracellular parts and also the outward tilt in TM5’s extracellular part, where the signal propagation starts. In our third restrained run (*rstr3*) which started with an alternative state of the receptor, directed closure of ICL3 was not observed. When initial and final snapshots were aligned, it was clear that the distance restraints forced TM3 rather than TM5 to be displaced and consequently, only a minor outward tilt in TM5’s upper half was observed with no significant displacement at its intracellular region. Addition of an extra restraint between backbone atoms of the same residue pair (S203-D113) produced the desired outward tilt of TM5 at the upper half in *rstr4* run. Consequently both TM5 and TM6 were displaced at the intracellular part and ICL3 closed instantaneously on G-protein binding site within 100 ns, which was the most rapid closure to be observed so far. Here, ICL3 adopted an alternative packed state, which was further observed to be stable for another 500 ns when the restraints were removed.

Another set of restraints that constrict the ligand-binding site was applied on the closed ICL3 state (*rstr5*) in order to free the loop from its interactions with the receptor. However, our attempt to open up ICL3 failed during the time scale of our runs. To summarize, our current study revealed alternative packed states of ICL3, which are stabilized by several hydrogen bonds between ICL3 and the rest of the receptor. Furthermore, in contrast to the persistence of ICL3 in its locked position, it was almost always straightforward to bring ICL3 from a loose, free state to a locked one by simply applying a few distance restraints that expand the extracellular ligand-binding site.

Starting with such *very inactive* states, the receptor stayed almost irreversibly inhibited during our runs, which in turn decreased the overall mobility of the receptor. Experimental support is currently lacking for the locked, inactive state of β_2_AR, due to the fact that ICL3 has been missing in most studies including the crystal structures.

The bond restraints imposed in our study simply represent the restrictions caused by ligands of various sizes bound at the ligand-binding site. Small agonist molecules tend to fit to a narrow region, whereas the antagonists and inverse agonists of larger size induce a more expanded binding site. As a result of allosteric coupling between intra- and extracellular regions, which is mediated through the transmembrane helices, particularly TM5 and TM6, the population of conformational states of ICL3 between unpacked and packed positions and thereby the binding of G-protein were modulated.

## Abbreviations

ANM, anisotropic network model; ECL, extracellular loop; GPCRs, G protein coupled receptors; ICL, intracellular loop; MD, molecular dynamics; PCA, principal component analysis; POPC, palmitoyl­oleoyl-phosphatidylcholine; RMSD, root mean square deviation; RMSF, root mean square fluctuation; T4L, T4-lysozyme; TM, transmembrane helix; TMEMB, transmembrane region; β_2_AR, β_2_-adrenergic receptor.

## Additional files

Additional file 1: Figure S1. Representations of (a) Core and (b) Tmemb regions highlighted in blue color in the receptor. (PDF 546 kb) Additional file 2: Figure S2. Root mean square deviations of the intracellular parts of TM5 (at the bottom) and TM6 (at the top) with respect to the active state (PDB id: 3P0G) for the original *1 μs* long MD run and two 500 ns long continuation runs (*MD1μs_ctd1* and *MD1μs_ctd2*). (PDF 28 kb) Additional file 3: Figure S3. Initial (in magenta) and intermediate (in cyan) snapshots of the original *1 μs* MD run from (a) side and (b) intracellular views. Here, the intermediate state was taken as the starting conformation for the third restrained run (*rstr3* in Table 2). (PDF 223 kb) Additional file 4: Figure S4. ICL3’s center of mass (*x* and *y* coordinates only) during the third 500 ns long restrained run (*rstr3* in Table 2). Stationary G protein’s α helix was also represented with lines connecting its *x* and *y* coordinates extracted from the active state’s crystal structure (PDB id: 3SN6). (PDF 110 kb) Additional file 5: Figure S5. Initial and final snapshots of the third 500 ns long restrained run *(rstr3* in Table 2). Top and bottom views are the extracellular and the intracellular sides, respectively. (PDF 1703 kb) Additional file 6: Figure S6. ICL3’s center of mass (*x* and *y* coordinates only) during the 8^th^ run (*MD500ns*), where all the restraints were released. Stationary G protein’s α helix was also represented with lines connecting its *x* and *y* coordinates extracted from the active state’s crystal structure (PDB id: 3SN6). (PDF 108 kb)
