# Supplementary figures and images for: Investigation of allosteric coupling in human β2-adrenergic receptor in the presence of intracellular loop 3
Source: BMC Struct Biol. 2016 Jul 2;16:9. doi: 10.1186/s12900-016-0061-9 (PMC4930610; doi:10.1186/s12900-016-0061-9)

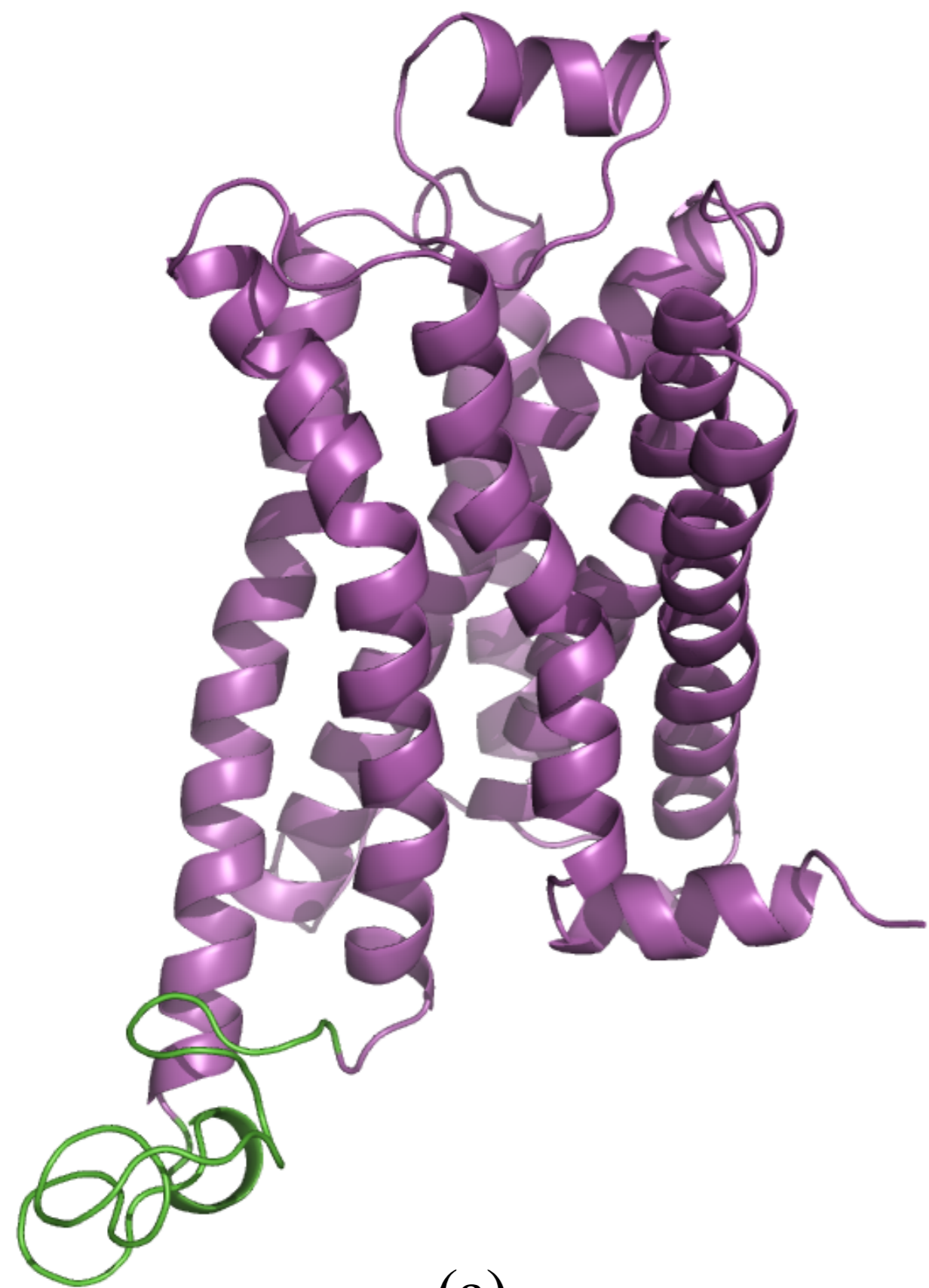

(a)

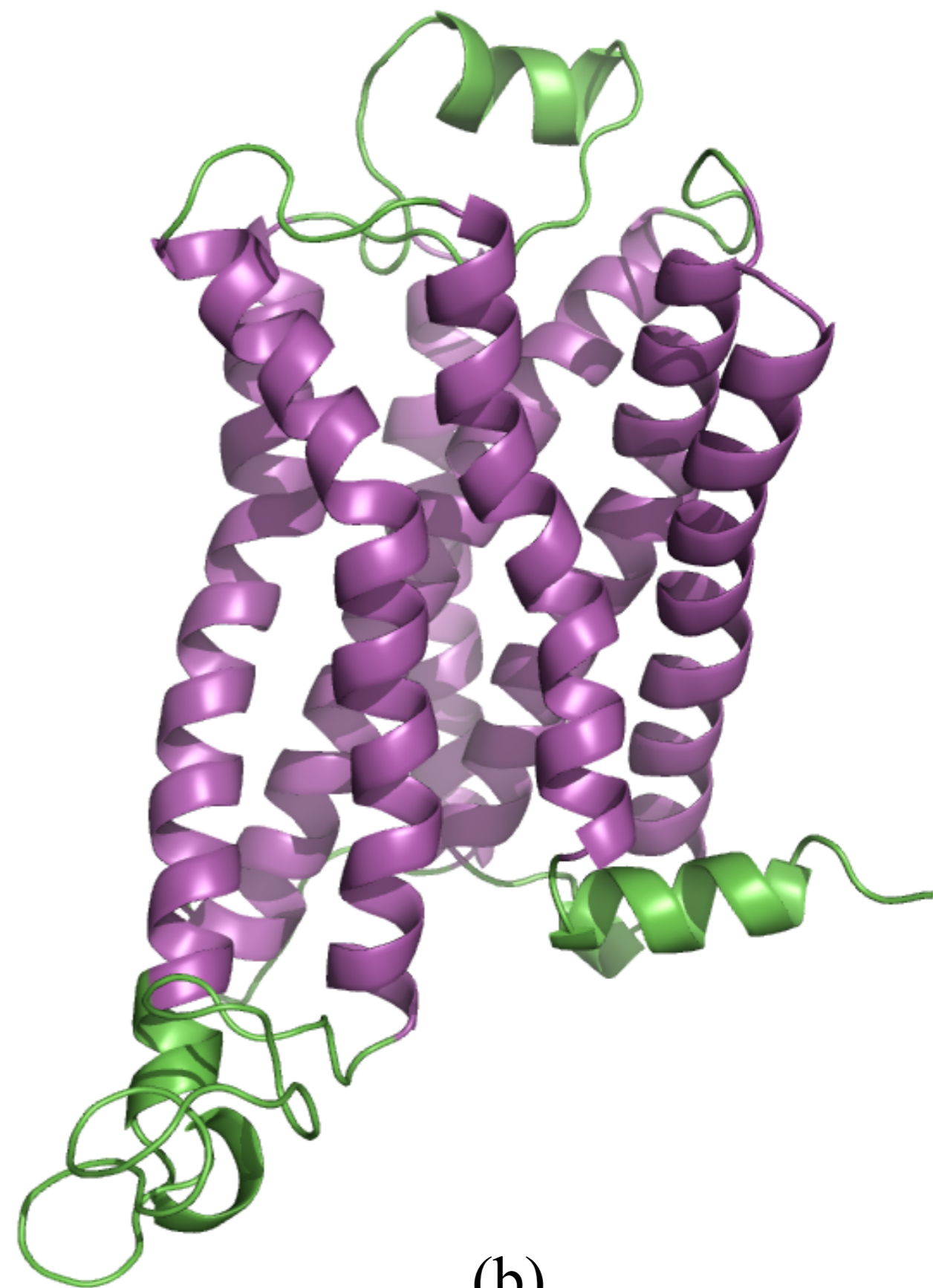

(b)

Supplement: Additional file 1: Figure S1. — Representations of (a) Core and (b) Tmemb regions highlighted in blue color in the receptor. (PDF 546 kb) [file 12900_2016_61_MOESM1_ESM.pdf]

RMSD (Å)

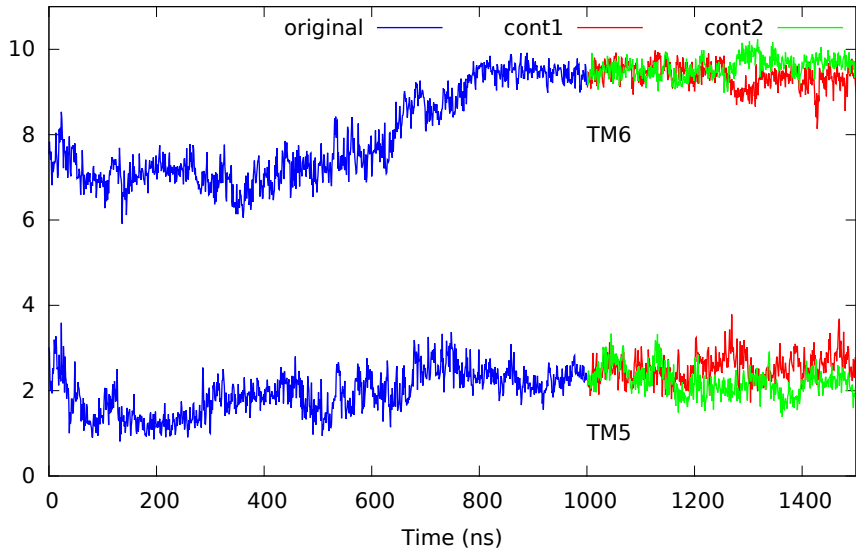

Supplement: Additional file 2: Figure S2. — Root mean square deviations of the intracellular parts of TM5 (at the bottom) and TM6 (at the top) with respect to the active state (PDB id: 3P0G) for the original 1 μs long MD run and two 500 ns long continuation runs (MD1μs_ctd1 and MD1μs_ctd2). (PDF 28 kb) [file 12900_2016_61_MOESM2_ESM.pdf]

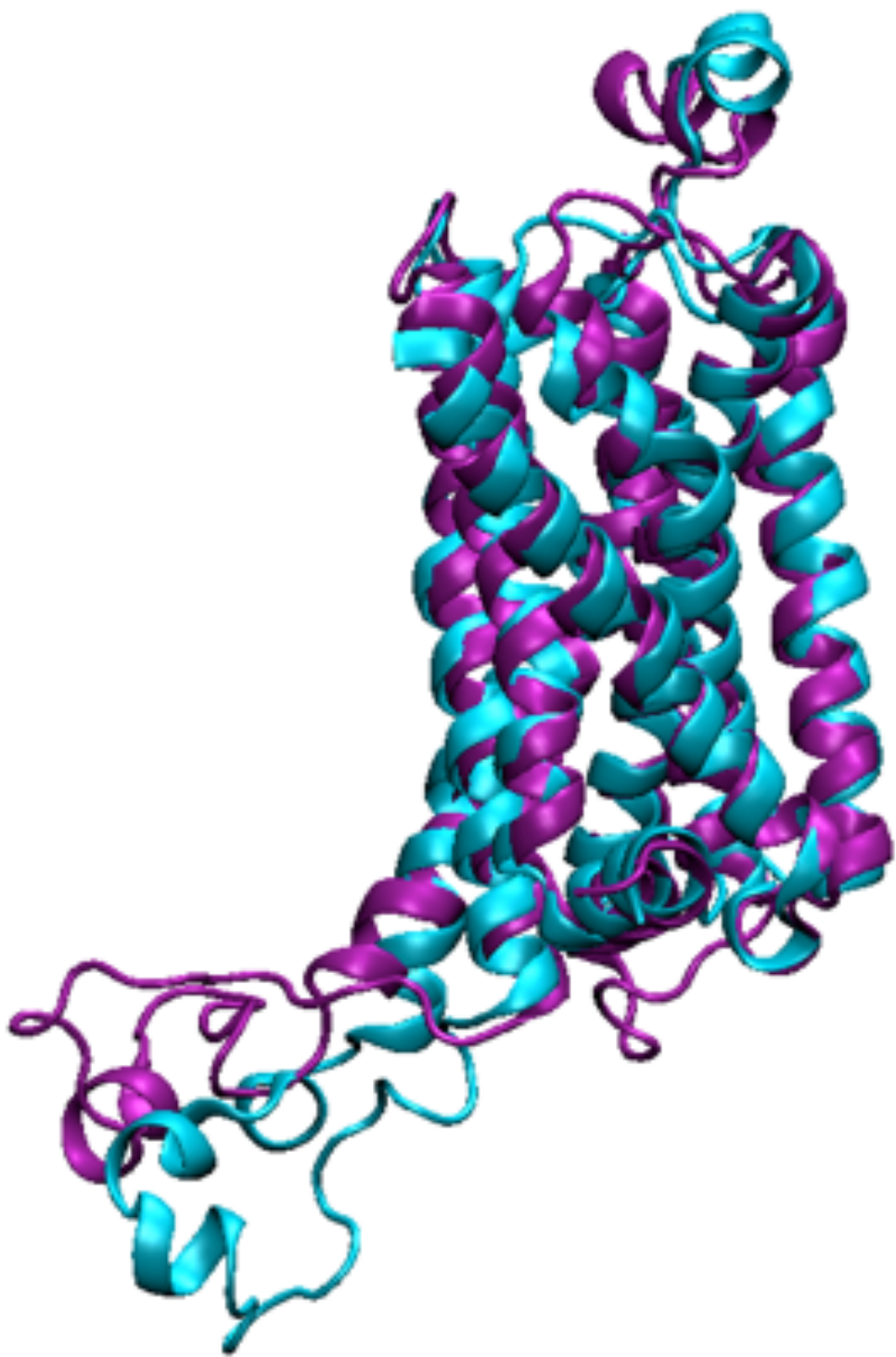

(a)

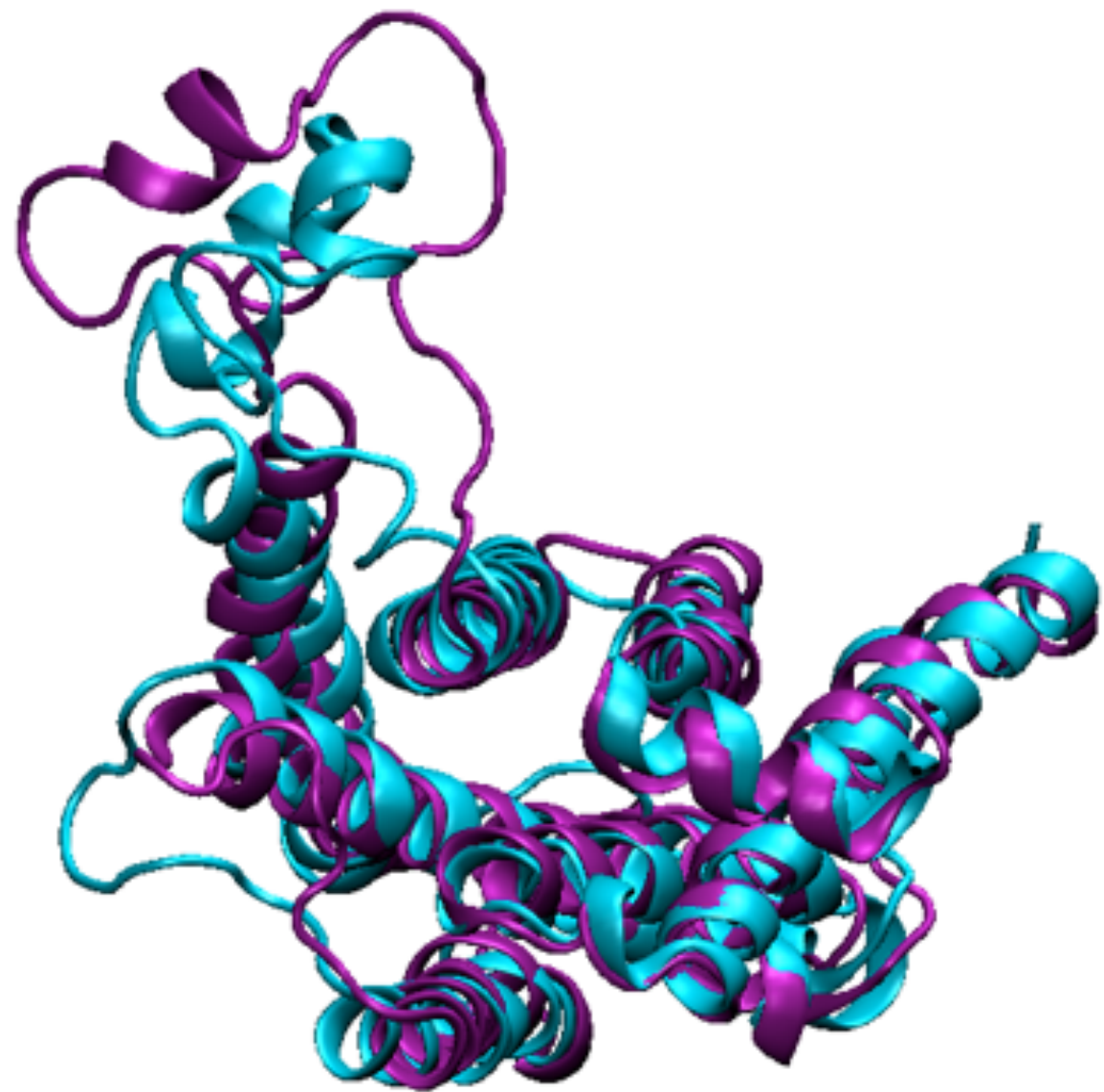

(b)

Supplement: Additional file 3: Figure S3. — Initial (in magenta) and intermediate (in cyan) snapshots of the original 1 μs MD run from (a) side and (b) intracellular views. Here, the intermediate state was taken as the starting conformation for the third restrained run (rstr3 in Table 2). (PDF 223 kb) [file 12900_2016_61_MOESM3_ESM.pdf]

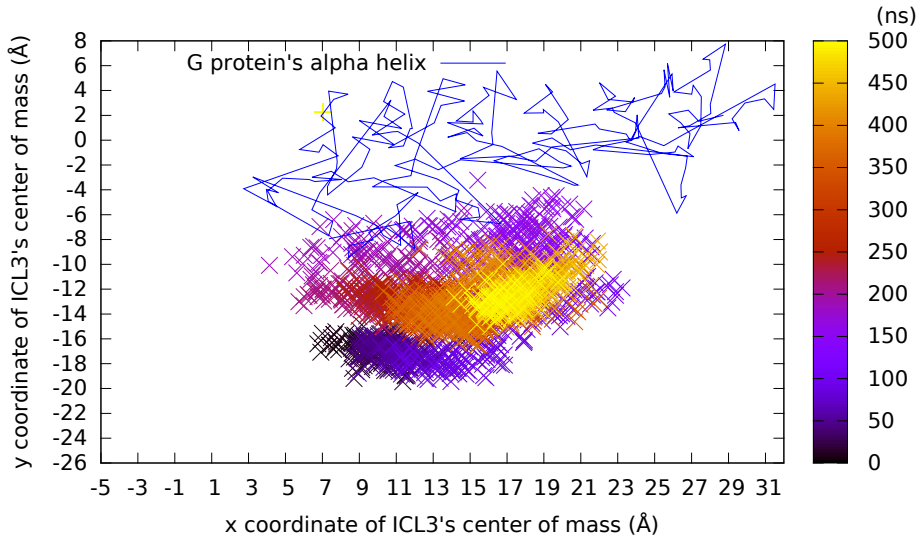

Supplement: Additional file 4: Figure S4. — ICL3’s center of mass (x and y coordinates only) during the third 500 ns long restrained run (rstr3 in Table 2). Stationary G protein’s α helix was also represented with lines connecting its x and y coordinates extracted from the active state’s crystal structure (PDB id: 3SN6). (PDF 110 kb) [file 12900_2016_61_MOESM4_ESM.pdf]

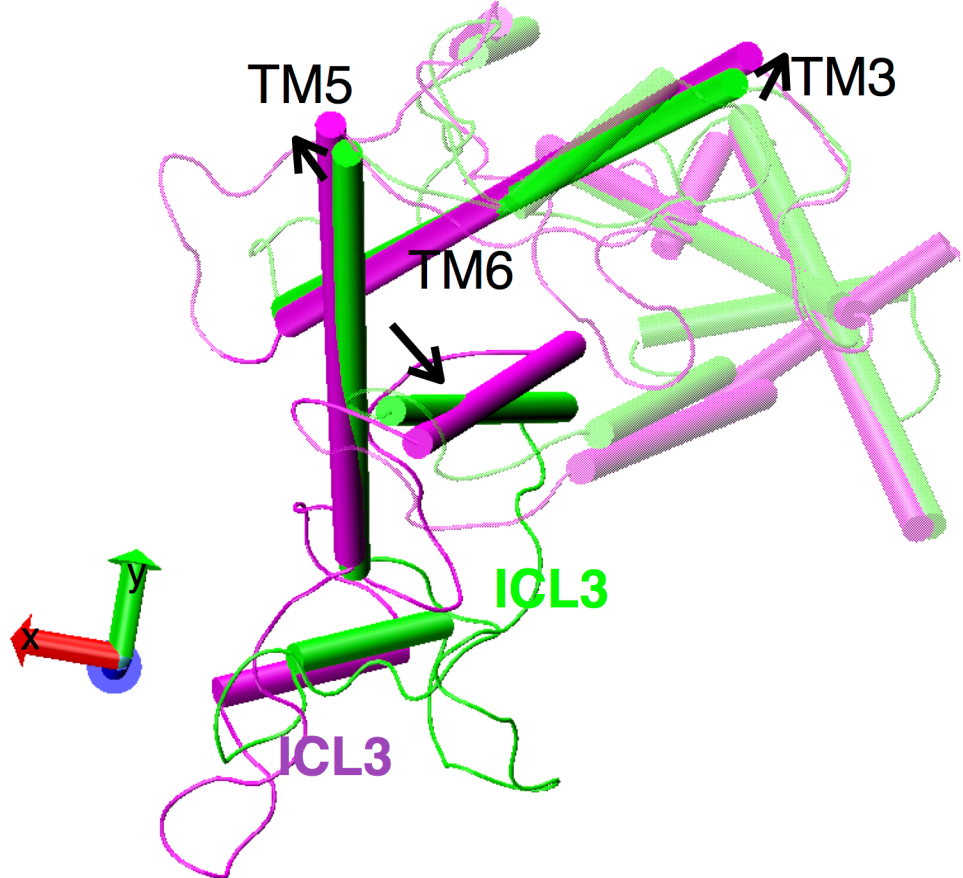

(a)

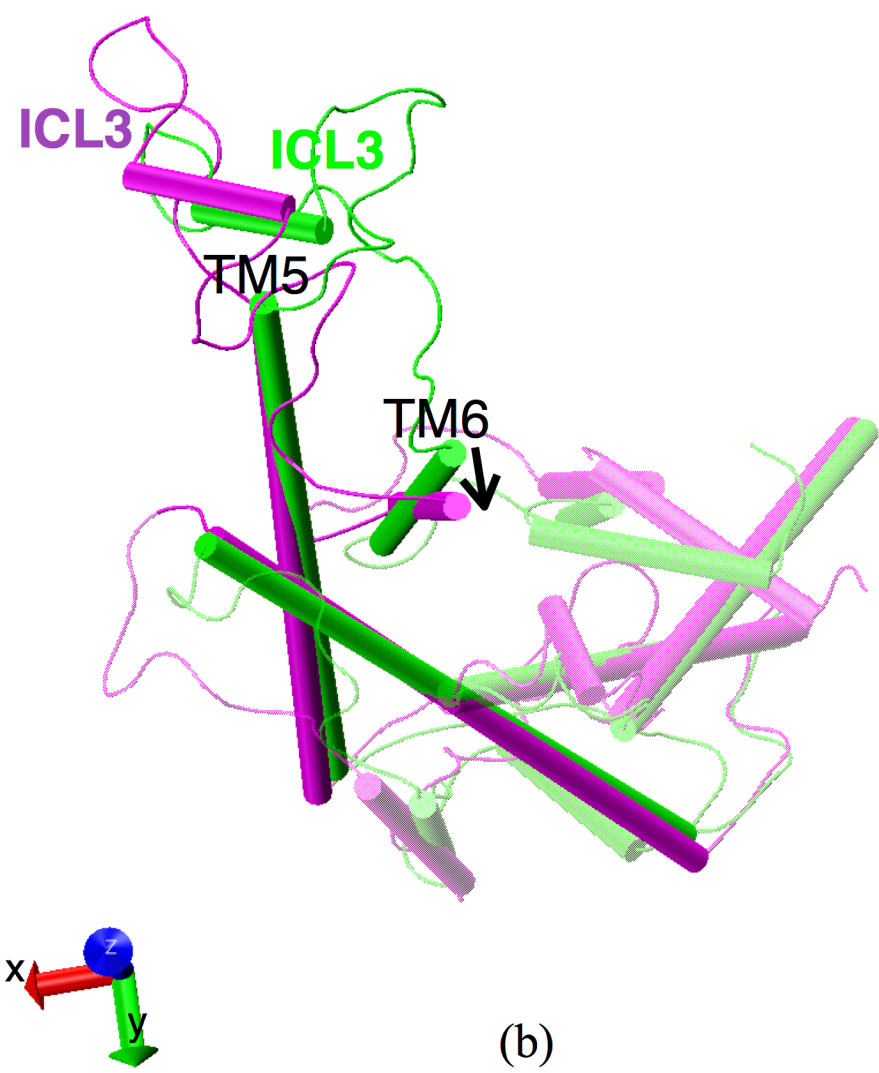

(b)

Supplement: Additional file 5: Figure S5. — Initial and final snapshots of the third 500 ns long restrained run (rstr3 in Table 2). Top and bottom views are the extracellular and the intracellular sides, respectively. (PDF 1703 kb) [file 12900_2016_61_MOESM5_ESM.pdf]

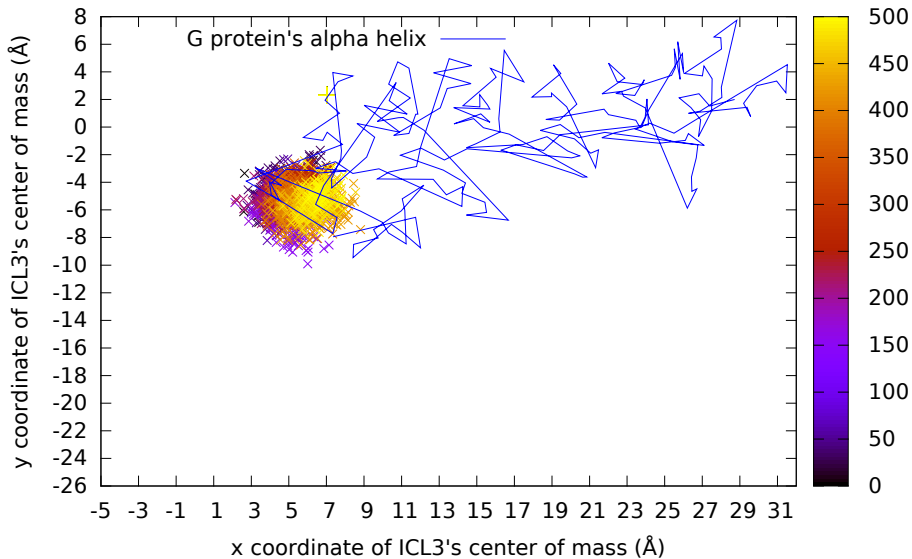

Supplement: Additional file 6: Figure S6. — ICL3’s center of mass (x and y coordinates only) during the 8th run (MD500ns), where all the restraints were released. Stationary G protein’s α helix was also represented with lines connecting its x and y coordinates extracted from the active state’s crystal structure (PDB id: 3SN6). (PDF 108 kb) [file 12900_2016_61_MOESM6_ESM.pdf]
